# An Analytical Model for Estimating Water Exchange Rate in White Matter Using Diffusion MRI

**DOI:** 10.1371/journal.pone.0095921

**Published:** 2014-05-16

**Authors:** Esmaeil Davoodi-Bojd, Michael Chopp, Hamid Soltanian-Zadeh, Shiyang Wang, Guangliang Ding, Quan Jiang

**Affiliations:** 1 School of Electrical and Computer Engineering, University of Tehran, Tehran, Iran; 2 Department of Neurology, Henry Ford Health System, Detroit, Michigan, United States of America; 3 Department of Physics, Oakland University, Rochester, Michigan, United States of America; 4 Image Analysis Laboratory, Department of Radiology, Henry Ford Hospital, Detroit, Michigan, United States of America; University of New Mexico, United States of America

## Abstract

Substantial effort is being expended on using micro-structural modeling of the white matter, with the goal of relating diffusion weighted magnetic resonance imaging (DWMRI) to the underlying structure of the tissue, such as axonal density. However, one of the important parameters affecting diffusion is the water exchange rate between the intra- and extra-axonal space, which has not been fully investigated and is a crucial marker of brain injury such as multiple sclerosis (MS), stroke, and traumatic brain injury (TBI). To our knowledge, there is no diffusion analytical model which includes the Water eXchange Rate (WXR) without the requirement of short gradient pulse (SGP) approximation. We therefore propose a new analytical model by deriving the diffusion signal for a permeable cylinder, assuming a clinically feasible pulse gradient spin echo (PGSE) sequence. Simulations based on Markov Random Walk confirm that the exchange parameter included in our model has a linear correlation (R^2^>0.88) with the actual WXR. Moreover, increasing WXR causes the estimated values of *diameter* and *volume fraction* of the cylinders to *increase* and *decrease*, respectively, which is consistent with our findings from histology measurements in tissues near TBI regions. This model was also applied to the diffusion signal acquired from *ex vivo* brains of 14 male (10 TBI and 4 normal) rats using hybrid diffusion imaging. The estimated values of axon diameter and axonal volume fraction are in agreement with their corresponding histological measurements in normal brains, with 0.96 intra-class correlation coefficient value resulting from consistency analysis. Moreover, a significant increase (*p* = 0.001) in WXR and diameter and decrease in axonal volume fraction in the TBI boundary were detected in the TBI rats compared with the normal rats.

## Introduction

Diffusion weighted imaging (DWI) is an interesting non-invasive tool for measuring micro-structural and physiological parameters of tissue. However, because of several limitations and restrictions in both imaging and methodology techniques, the task of relating specific microstructures to the diffusion signal is not straightforward and requires many simplifying assumptions. On the other hand, for measuring and estimating micro-structural and physiological parameters of the tissue, such as cell size and water exchange rate between intra- and extra-cellular spaces, high b-value diffusion imaging is generally required [1].

Currently, high b-value diffusion analysis has mainly focused on free model approaches to resolve intra-voxel fiber crossing, such as diffusion spectrum imaging (DSI) [2], high angular diffusion imaging (HARDI) [3], Q-ball imaging [4], and persistent angular structure MRI (PASMRI) [5].

In contrast, there are also some high b-value diffusion approaches in which a model is constructed based on the tissue structure and physical rules such as Fick’s laws to simulate diffusion and investigate its behavior under different conditions. Solving Fick’s laws even in a simple tissue model requires extensive calculations. In these cases, Fick’s law is solved using numerical techniques such as finite element [6], finite difference [7], [8], or partial differential equations (PDE) [9]. For complicated structures, probabilistic methods like Markov Random Walks (MRW) may be used [10]. After simulating the water diffusion in the structure, it is possible to calculate the diffusion signal using proper mathematical relations. Thus, these models are good validation tools in building a physical model by ensuring that each of its parameters has an obvious effect on the diffusion signal [11], [12]. Moreover, they can be used to estimate unknown parameters of the tissue. For example, the conventional DTI model combined with a physical simulation model of axons [13] is used to estimate white matter tissue parameters such as density and myelin sheath thickness [14].

Other approaches utilize and optimize the parameters of the MRI pulse sequence in order to make it sensitive to specific microstructural parameters [15]. For example, double pulsed-field gradient (d-PFG) MRI scheme is used to measure pore diameter [16] and double-wave-vector imaging protocol is used to estimate the density and size of cells even in irregular distributions [17]. The phase of the diffusion signal also contains useful information about the tissue such as fiber orientation [18]. On the other hand, high field diffusion imaging is also an interesting tool to measure cell size because of the diffraction patterns seen in the diffusion signal in high field gradients [19]. This approach is employed to measure the size of microstructures in a porous medium [20], [21]. However, it hardly can be applied to white matter fibers due to its limitations in both imaging and modeling methodologies [21]. Another example of high field imaging application is the work of Jespersen et al. [22] in which a two-compartment diffusion model is proposed to measure the density of neurites (axons and dendrites) for a normal rat.

Presently, most researchers use muli-compartment models for modeling of diffusion signal acqired by MRI [1], [23]–[25]. Typically, they consist of at least two compartments: one compartment is devoted to diffusion in the extra-cellular or extra-axonal space, known as hindered diffusion or fast diffusion. The other part is diffusion in the intra-cellular or axonal space, known as restricted diffusion or slow diffusion. Different approaches define and use different parameters and different simplifying assumptions. The CHARMED (Composite Hindered And Restricted ModEl of Diffusion) introduced by Assaf et al. [1] is a well known model that has been used in several studies to measure tissue parameters, especially axon diameter. For example, Assaf et al. [23] estimated axon diameter distribution of freshly excised porcine optic and sciatic nerves using this model and high b-value MRI perpendicular to the axons. However, one of the first attempts to measure axon diameter distribution *in vivo* was conducted by Barazany et al. [25] for the corpus callosum fiber tracts of rats by combining DW imaging and a new analytical method called AxCaliber proposed by Assaf et al. [24]. This work is an extension of the CHARMED model which has an additional compartment for isotropic diffusion, accounting for cerebrospinal fluid (CSF). In this approach, the diffusion gradients also need to be perpendicular to the fiber orientation. To reduce the dependence on the fiber orientation, Alexander et al. [26] proposed a four compartment model consisting of intra-axonal space, extra-axonal space, CSF, and glial cells combined with a multi-shell HARDI data acquisition. The scalars defined in this method for estimating the axon diameter and density are invariant to the orientation of the axons. Another limitation of this kind of modeling is that a single orientation for the axons is assumed which limits the analysis to the voxels belonging to single fibers. Therefore, Zhang et al. [27], using a simplified version of the CHARMED model, brought in the effect of orientation dispersion of the axons to the modeling by assuming Watson distribution for the axon's orientation. Furthermore, by modifying their model they [28] proposed a three comportment model combined with a clinically feasible two-shell HARDI protocol to estimate the neurite orientation dispersion and density (NODDI). Hence, this approach can be applied to a wide range of voxels within the brain. As a final point here, Panagiotaki et al. [29] did an experimental study to investigate the performance of 47 multi-compartment models of the white matter with no exchange. They concluded that by combining a diffusion tensor (DT), a cylindrical, and a spherical component, the constructed model describes the data more accurately. Moreover, considering a distribution rather than a single value for the axon’s radius decreases the stability of the fitting process.

It should be mentioned here that *Ball* and *stick* [30] is another kind of modeling which consists of two terms: one for modeling the isotropic diffusion, *ball*, and the other is accounting for the fibers, *zero-radius stick(s)*. Although this kind of modeling looks similar to two compartment models like CHARMED, it is mainly useful for resolving the fiber orientation in crossing regions.

An interesting parameter of the white matter is the water exchange rate between the intra- and extra-axonal spaces, which also has an effect on the diffusion signal [31]. Karger et al. [32] added the exchange effect into the NMR magnetization differential equations to derive proper relations to measure exchange times. One of the first analytical models including exchange rate was introduced by [33] for the bovine optic nerve using Karger relations. There are also other studies which investigated the effect of the exchange rate based on the Karger framework [12], [31], [34], [35]. Injuries or abnormalities of the cerebral tissues can change the exchange rate [35]. Moreover, it is shown using Monte Carlo simulation that in a two-compartment diffusion model, the exchange rate through a permeable membrane should be included so that the estimated parameters are close to their actual values [36]. Usually, these models are valid when the diffusion gradient is applied perpendicular to the tract. The short gradient pulse (SGP) condition also needs to be valid when applying the NMR imaging protocol. On the other hand, some techniques modify and optimize the acquisition pulse sequence to make it sensitive to the exchange rate, such as double-PGSE diffusion exchange spectroscopy [37], filter exchange spectroscopy (FEXSY) [38], and apparent exchange rate (AXR) imaging [39].

Another way to import the exchange effect into the modeling of the diffusion signal is to solve Fick’s Laws for a typical structure with proper boundary conditions, which considers permeable membranes [40]. However, even for an impermeable boundary, solving Fick’s equations is difficult and needs simplifying assumptions such as SGP or identical diffusion time, Δ, and diffusion pulse width, δ, [41] which limit the applicability of the methodology for a typical human MRI scanner.

Therefore, the aim of this work is to present a new analytical model for the diffusion signal which includes axon diameter and axonal volume fraction as well as water exchange rate. The diffusion signal is calculated mathematically for a pack of parallel cylinders with permeable membranes, assuming a practical PGSE sequence. The model has been validated and confirmed using artificial data with Markov Random Walk simulation, DWMRI, and histological data from the *ex vivo* rat brain. The model is also used to compare TBI rats with normal ones with respect to the estimated parameters, especially in the corpus callosum.

## Materials and Methods

In this section, we derive the diffusion signal assuming a PGSE sequence perpendicular to a typical cylinder having permeable membrane. Then the other parts of the model, accounting for the diffusion signal of parallel and extra axonal spaces, are combined accordingly. After that, the methodology for evaluating the proposed model using artificial data simulated by Markov Random Walk is described. Finally, the DWI protocol and the staining process employed in our experiments are explained.

### a) Background and Motivation

The general equation for relating the diffusion signal to the movement of the spins during diffusion time, Δ, is
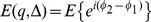
(1)in which (*φ_2_*−*φ_1_*) is the phase change due to the spin displacement along the direction of the diffusion gradient. To solve equation (1), the probability function of the phase change has to be known. The simplest way is to assume a Gaussian distribution for this function; therefore we will have



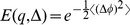
(2)This assumption is valid when diffusion is un-bounded or free in the underlying medium like inside the extra-cellular space. However, usually the researchers use the above equation also for bounded media like the axons in order to simplify the problem [41]. Under this assumption, the problem is reduced to calculate the “mean square phase change due to the spin displacement”, i.e.,
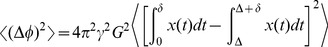
(3)in which *γ* is the gyromagnetic ratio, δ and Δ are the pulse width and diffusion time, respectively. Moreover, G is the amplitude of the diffusion gradient, which we assumed along the x-axis here. This also requires the probability function (propagator) of spin displacement, *P(r|r')*, which comes from solving Fick’s second law under proper initial and boundary conditions consistent with the underlying structure.

Another way to solve equation (1) is to assume δ<<Δ (short gradient pulse, SGP, approximation) and then use the Fourier representation of the problem [42]. Therefore, the Gaussian assumption for phase change does not have to be imposed. However, the propagator function *P(r|r')* is still required.

SGP approximation can hardly be achieved in existing clinical and even research MRI scanners. On the other hand, the first method imposes the Gaussian distribution on the model, which restricts the number of unknown parameters. For example, Neuman [41] calculated the perpendicular part of intra-axonal diffusion signal as
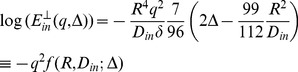
(4)where *q* = *γGδ.* Here, just one unknown parameter can be estimated if the images are acquired using a fixed value of Δ. For instance, in the CHARMED model [1], only the radius, *R*, is estimated using this part, while *D_in_* is estimated using the parallel part of the signal. To add another parameter to the model, such as exchange rate, there are two options; either we can define and use a new imaging protocol with different diffusion times, like [37], or we can build our model in a way that does not require Gaussian assumption for phase change and also does not require SGP approximation. Although the second approach is more realistic, to our knowledge, there is no calculation trying to use it because it necessitates non-solvable integrals. Here, we built an analytical model including a parameter accounting for the exchange rate using proper mathematical relations without assuming Gaussian distribution for the phase change or the SGP approximation.

### b) Proposed Model

The phase change can be approximated by the summation instead of the integral:
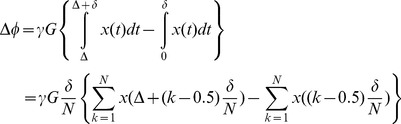
(5)where N>>1. Assuming diffusion gradient is along the x-axis, the more general form will be
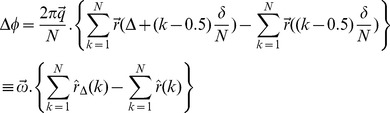
(6)where, 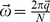, 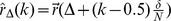, and 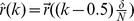.

Then, using equation (1), the diffusion signal is calculated by the following integral:
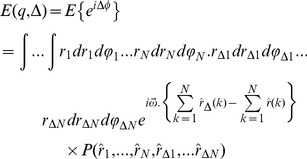
(7)in which 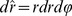. Since the diffusion is a Markov random process, each movement of a spin depends only on its starting position. Therefore, after replacing 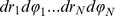 by 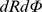 and expanding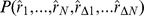, equation (7) is simplified to:



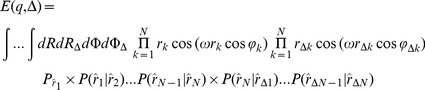
(8)Note that the imaginary part of equation (7) is equal to zero.

Now we need the probability function (propagator) of spin displacement, *P(r_0_|r)*. This function has been calculated for planar, spherical, and cylindrical boundary conditions [41], [42] and used in several calculations. However, few of them consider the effect of permeable membrane in the boundary condition [40]. For a cylinder of radius *R*, the 1-D propagator function perpendicular to the axon is [42]:
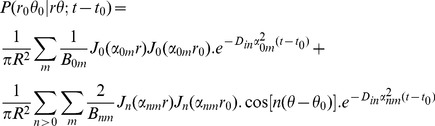
(9)which is the solution of the Fick’s Law,



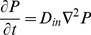
(10)subject to the following *initial* and *partially permeable membrane* boundary conditions:
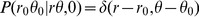
(11)

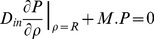
(12)where *D_in_* is the diffusion coefficient of the intra-axonal space and *M*(*m*/*s*) is the permeability coefficient. *J_n_*(.) is the *n^th^* order Bessel function and *α_nm_* is the *m^th^* positive root of the following equation which comes from combining equations (10) and (12).
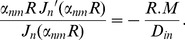
(13)The right hand side of the above equation is a dimensionless quantity which we call *absorbing coefficient*, *h* = *R*.*M*/*D_in_*. This quantity is directly related to the *exchange rate* (1/*s*), defined as the fraction of spins exchanged between intra- and extra- axonal space through the membrane per second, because each spin absorbed by the membrane moves to the extra- axonal space and there will be also a corresponding spin which is transported into the intra- axonal space in order to preserve the equilibrium. The coefficients *B_nm_* can be derived using the orthogonality property of the Bessel Functions and the above boundary condition:



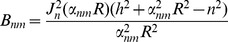
(14)By solving equations (8), (9), (13) and (14), the intra-axonal compartment of the diffusion signal perpendicular to the axon is achieved. Starting from equation (8), by taking the integrals over angels, *φ*, using the following equation
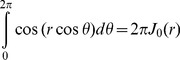
(15)


the equation (8) is reduced to:
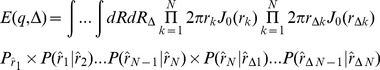
(16)in which 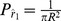 and the other probability functions are derived from equation (9). The most dominant term in that equation is the one having *α_01_* [40], [43], therefore,



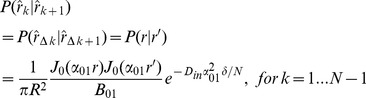
(17)

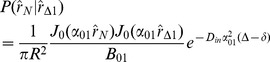
 (18)

where
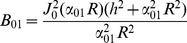
(19)and the simplified boundary condition will be
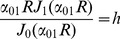
(20)


After inserting (17) and (18) into (16), we have
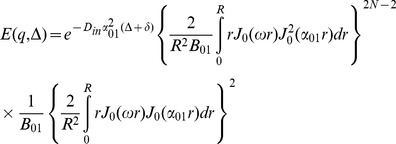
(21)


Unfortunately, only the second integral has a closed form which is
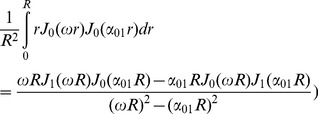
(22)


To get a closed form for the first integral, we assume that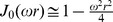 which is valid because *N* is considered large enough in our analysis. Using this approximation and also the following integral equation,
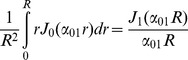
(23)


the closed form of the first integral appeared in equation (21) will be
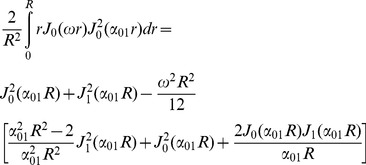
(24)


Finally, by simplifying equation (21) using equations (20), (22), and (24) we have
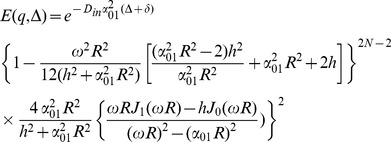
(25)


Therefore, the final form of the signal which we use in our model is:
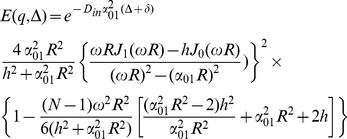
(26)where *α_01_* and *h* are related by equation (23).Here we should note again that in equation (9) we omit the terms with *n*>0 and *m*>1 from the summation as also assumed by [40], [43]. From equation (13) it can be shown that *α_nm_R*≥3.83 for all values of *n* and *m* except (*n*,*m*) = (0,1). Therefore, to ensure that this approximation is valid, we should have:



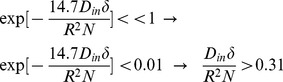



Thus, there is an upper limit for *N*, which is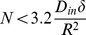. Combining this limit with the lower limit introduced in equation (5), we have:
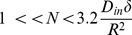
(27)


For example, since in our experiments, the typical values of *D_in_*, *R*, and δ are 0.25 µm^2^/ms, 0.5 µm, and 10 ms, respectively, a reasonable value of *N* is 30 which is chosen in our analysis.

For special cases when the SGP approximation is valid, i.e., δ<<1, we can set *N* equal to 1. Then, equation (26) is reduced to the following form of the diffusion signal, which is similar to the form used by [40]:
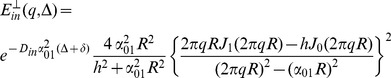
(28)


Moreover, if the absorbing effect, *h*, (or the exchange phenomenon) is neglected from the equation, we have:
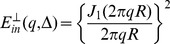
(29)


which is consistent with the form of the signal introduced by [43], for long Δ.

Similar to [1], if the diffusion gradient, *G*, is decomposed into parallel and transverse directions of the axon, i.e., *G_||_* and *G_⊥_*, and since *q* = *γδG,* the attenuated signals due to diffusion *in* and *out* of the axons are as follows
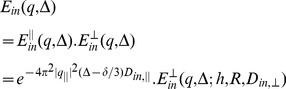
(30)

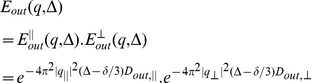
(31)


assuming Gaussian profile for *E_in_* ^||^, *E_out_^|^* ^|^, and *E_out⊥_*. The overall diffusion signal is a weighted summation of both compartments based on their volume ratios:
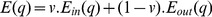
(32)


in which *v* is the intra-axonal volume fraction ratio. Also, if the Johnson noise model is considered for the measurements [1], the final model for the measured diffusion signal is.
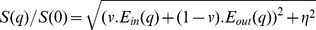
(33)


Assuming *D_in,||_* = *D_in,⊥_* = *D_in_*, there are totally 10 parameters in our model: Diffusion coefficients (*D_in,||_*, *D_out,||_*, *D_out,⊥_*), axon radius (*R*), absorbing coefficient (*h*), axon direction (*n*), volume fraction of axons (*v*), and also noise level (*η*).

In summary, our two compartment model consists of four parts to model the diffusion signal. Among these parts, the effect of membrane permeability is modeled explicitly only by *E_in⊥_* which takes into account the spins inside of the axon absorbed by the membrane. This means that those exchanging spins which leave or enter the intra- axonal space are modeled by the other parts which assumed to have Gaussian distribution. As a result, this assumption may affect the estimation of the other parameters of the model especially the apparent diffusion coefficients as in current non-exchanging two compartment models. However, as stated before, the absorbing coefficient, *h*, included in the proposed model is a quantity which can be used to estimate the exchange rate.

### c) Model Fitting

**Figure 1:**
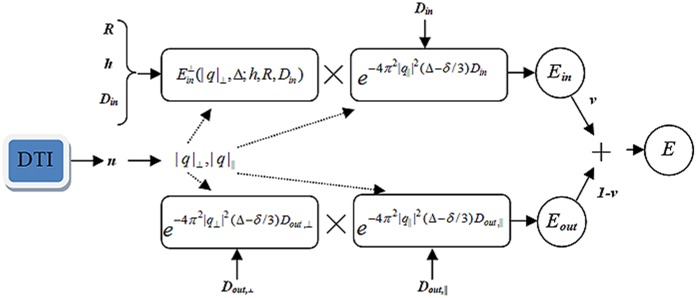
The overall look of the proposed model for deriving the diffusion signal. The unknown parameters are *D_in_*, *D_out,||_*, *D_out,⊥_*, *R*, *v*, and *h*. The direction of axons is estimated using the conventional DTI model.

Having measurements and the proposed model, the remaining task is to fit the model to the data. In this work, we use a non-linear least square fitting procedure to estimate unknown parameters (*D_in_*, *D_out,||_*, *D_out,⊥_*, *R*, *h*, *v*, and *η*). For the fiber direction, we use the principal eigen-vector estimated from the DTI model. Note that since the proposed model is built for a specific configuration of fibers (parallel axons with nearly identical diameters), it is valid just for a special group of voxels. Therefore, we apply the model to the voxels having high FA values which are more likely to belong to a single fiber tract. We also assume that in a single fiber, the axons are parallel and nearly the same size. The fitting procedure is implemented in MATLAB (MathWorks, Natick, Massachusetts, USA) by employing its optimization toolbox. A block diagram of the proposed model for driving the diffusion signal is shown in Figure 1.

### d) Markov Random Walk Simulation

Validation of a model can be conducted using simulation data. In this work, we use Markov Random Walk (MRW) to simulate the diffusion process and create an artificial diffusion signal in a voxel. An advantage of MRW is that it can be applied even to complex configurations of the fibers. Nevertheless, a disadvantage of MRW is its huge computational complexity. The aim of this simulation is to solve the diffusion equation for a fiber.

**Figure 2:**
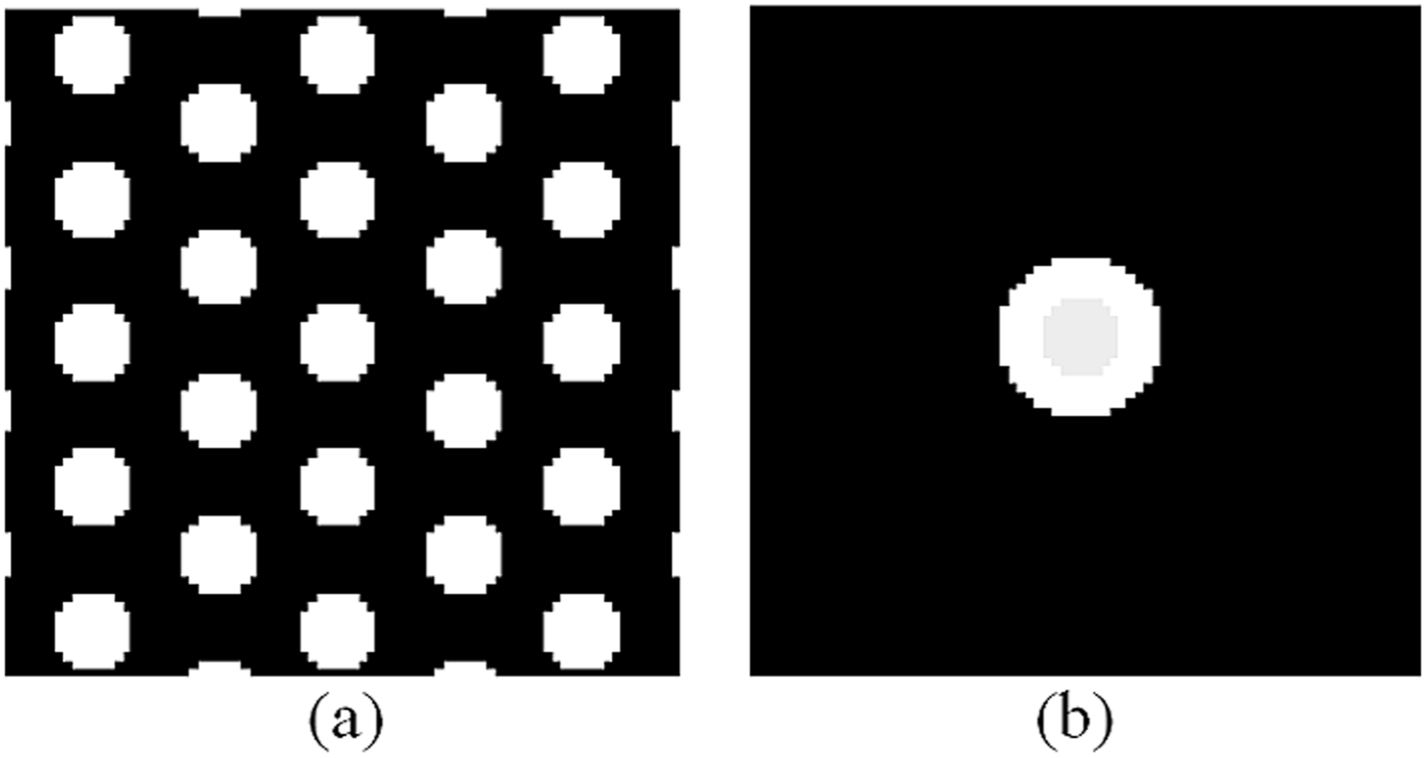
Cross sectional view of the distribution of (a) the axons and (b) the spins at *t* = 0. The axons are realized as parallel same size cylinders with permeable boundaries. Initial spins are distributed in the center of the voxel such that the ratio of spins inside the central axon is equal to the axonal volume fraction.

Consider a pack of parallel cylinders of equal radius, *R*, (rolling as axons) distributed in a regular manner (see Figure 2(a)). Thus, the medium is partitioned into two components: intra- and extra-axonal spaces having the diffusion coefficients of *D_in_* and *D_out_*, respectively. To simulate diffusion, first we construct a 3D grid of cells to discretize the space. In this work, we assume a voxel with dimension of 24×24×48 µm discretized (separated) into 80×80×160 cells (Δ*x* = Δ*y* = Δ*z* = 0.3 µm). Axons are considered as parallel cylinders in the z-direction with a radius of 1.5 µ*m*. Axonal volume fraction, *v*, is defined as the ratio of the intra-axonal space to the total volume. In this work, we assume two configurations with *v* = 0.27 and *v* = 0.46.

To simulate the random walk, each spin starts from its initial position and moves with a step size, *dr = √(D.dt/6)*, in one of the 6 directions along the Cartesian axes, i.e., −x, x, −y, y, −z, z, randomly. Note that the step size, *dr*, is different from the cell size, Δ*x*, Δ*y*, or Δ*z*. So, based on its position and the diffusion gradient’s direction and magnitude, the phase of that spin is updated by.
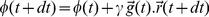
(34)


with the initial value of *φ*(*t* = 0) = 0 and time step, *dt*, equal to 2 µs. Then, the attenuated signal due to diffusion at echo time, *t* = TE, is calculated using the following relation:
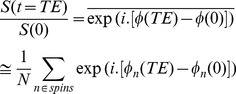
(35)


**Figure 3:**
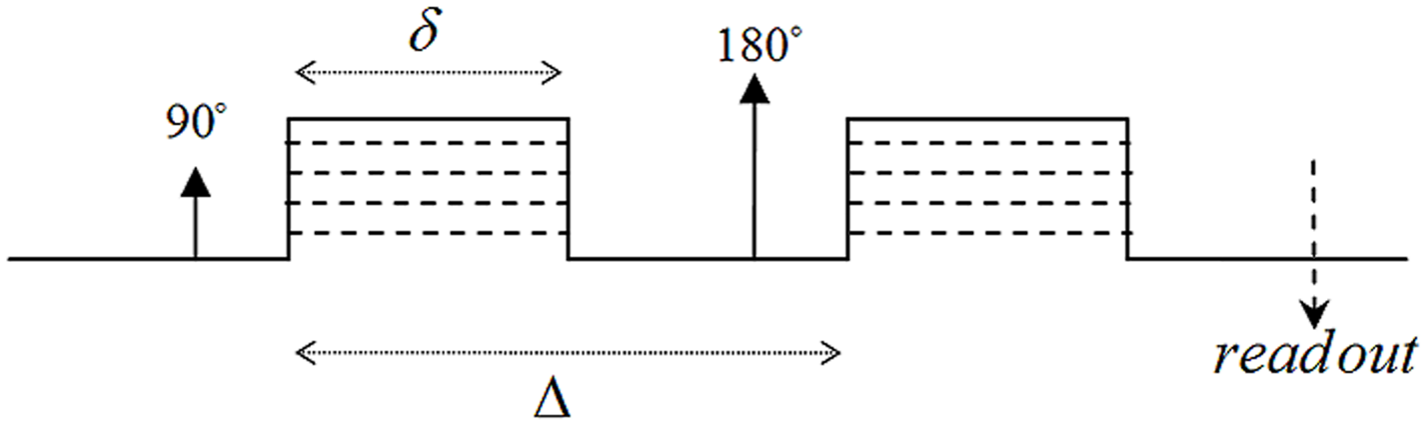
A typical PGSE sequence. δ is the gradient pulse width and Δ is the diffusion time.

At *t* = 0, 10^6^ spins are distributed uniformly on a disk which is placed in the center of the voxel (see Figure 2(b)). The radius of the disk is selected such that the ratio of the initial spins inside the cylinder to the outside ones is equal to the intra- to extra-axonal volume ratio. We should emphasis that it is not necessary to initialize the spins across the whole substrate because in our simulation the configuration is symmetric. Moreover, in this way, the likelihood that a spin reaches to the boundary of the voxel is significantly decreased which is desirable in the numerical calculations. To calculate the diffusion signal, we consider a typical gradient spin echo sequence as depicted in Figure 3. The sequence parameters are chosen similar to our *ex-vivo* animal experiment with δ = 10 *ms*, Δ = 18 *ms*, and *q* = 0.025, 0.05, 0.075, 0.1, 0.125 1/*µm,* corresponding to 357, 1437, 3240, 5767, 9016 *s*/*mm^2^* b-values, respectively. However, the number of diffusion gradients is set to 6 for all five shells to reduce computation time.

**Figure 4:**
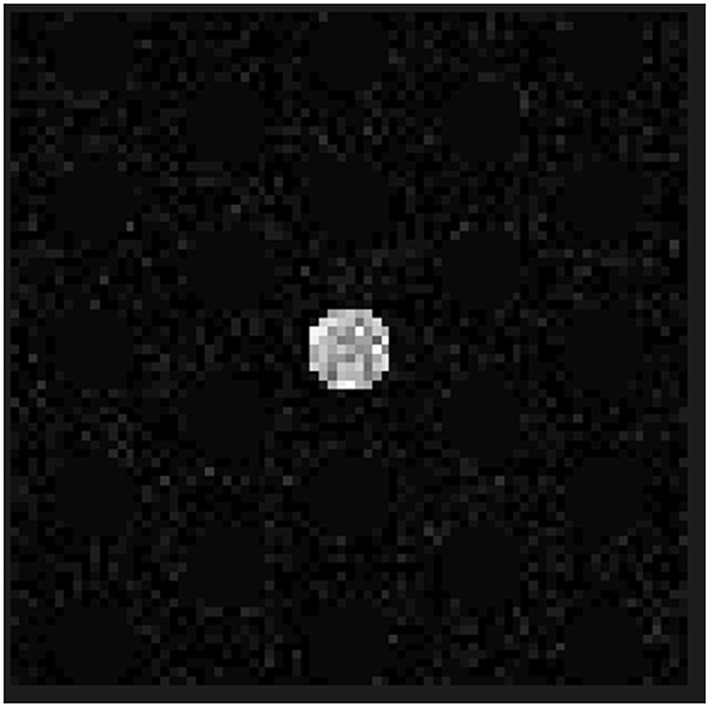
A typical result of final distribution of the particles shown in a cross section of the structure. In this experiment, the particles did not pass the boundaries because permeability was set to 0.

To include the exchange process into the simulation, the spins reaching the boundary may pass it, based on a jump probability, *s*, which is calculated from the following equation,
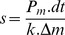
(36)in which *P_m_* is the membrane permeability, Δ*m* is the membrane thickness, and *k* represents the relative concentration of the inside/outside of the axon to the free water which we set it to 1 in this work. As suggested by Hwang et al., 2003, we set the membrane thickness equal to 0.01 *µm*. Also, we choose different values of 0,1,2,3,4,5,6,9,12 *µm*/*s* for the permeability to investigate the effect of exchange on the signal. At the end of each experiment, *Water Exchange Rate* (WXR), 1/sec, is calculated as the ratio of the spins passed the boundary to the total number of initial spins per second. Moreover, to study the performance of the proposed model more realistically, white Gaussian noise is added to the signals (SNR = 40) and each investigation is repeated 50 times. In Figure 4, the final distribution of the particles is shown in a cross section of the structure. In this simulation, the permeability is equal to zero; therefore, the particles did not pass the boundaries.

**Figure 5:**
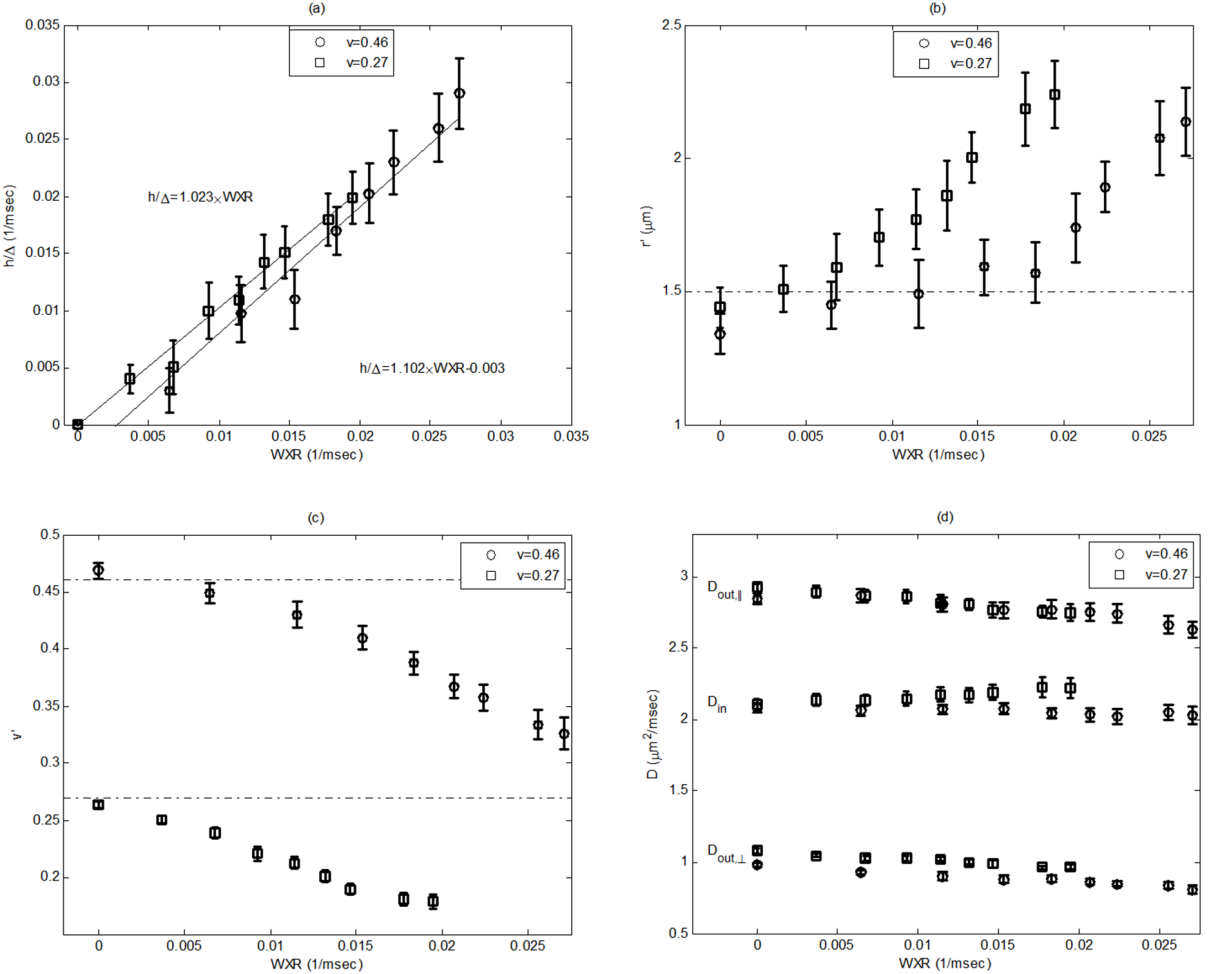
Error bar plots of the mean and STD values for the estimated parameters (a) *h/Δ*, (b) *r’*, (c) *v’*, and (d) diffusion coefficients. *h/Δ* has a significant (*p* = 000.1) linear correlation to WXR, with R^2^-values of 0.91 and 0.88 for *v* = 0.46 and *v* = 0.27, respectively. Also, the intra-class correlation analysis within the 95% confidence interval shows 0.968 and 0.967 agreement (1∶1 match) (p = 0001) between *h/Δ* and WXR for these two configurations, respectively. It is also seen that the diameter increases while the axonal fraction decreases as the WXR increases.

In Figure 5, the mean and STD values for the estimated parameters *h/Δ, r′*, *v′*, and the diffusion coefficients are shown versus WXR. Note that *h/Δ* is the absorbing coefficient normalized to the diffusion time. It can be seen that in Figure 5 (a) *h/Δ* has a significant (*p* = 0.0001) linear correlation to the WXR, with R^2^-values of 0.91 and 0.88 for *v* = 0.46 and *v* = 0.27, respectively. Also, the intra-class correlation analysis within 95% confidence interval shows 0.968 and 0.967 agreement (1∶1 match) (*p* = 0.0001) between *h/Δ* and WXR for these two configurations, respectively. On the other hand, the diffusion coefficients have also been well estimated using the model. However, for radius, *r*, and volume fraction, *v*, the estimated values change as the WXR increases. Note that *v′* actually corresponds to the ratio of spins trapped inside axons, which decreases by increasing WXR. On the other hand, by decreasing *v′,* one may expect that the radius should decrease too. However, it should be noted that in our model, the number and geometrical configuration of the axons is not considered. Therefore, increasing the radius does not necessitate increasing the intra-axonal space. Moreover, one reason the estimated radius, *r′*, is increased by increasing WXR is that when more spins pass the axon’s boundary, the effective apparent radius increases. It should be stated that compensating for these two effects is almost impossible using two-compartment models. However, it may be solved if the number and geometrical configuration of the axons are known and imposed into the model using proper relations.

### e) Experimental Design

To estimate unknown parameters using the proposed model, there should be appropriate diffusion MRI data. First, data should be acquired using more than one b-value (multi-shell diffusion imaging) so that the profile of diffusion in the radial directions can be revealed. Secondly, the diffusion time, Δ, should be large enough so that the spins have enough time to reach the boundaries.

#### 1) Animal preparation

### All Experimental Procedures Were Approved by the Institutional Animal Care and Use Committee (IACUC) of Henry Ford Health System

Fourteen male Wistar rats (weight: 270–300 g) were divided into two groups, traumatic brain injury (TBI) (*n* = 10) and a normal group without neurological injuries (normal group, *n* = 4). All TBI rats were subjected to controlled cortical impact (CCI) [44] and sacrificed six weeks after TBI. The brain was perfused with heparinized saline [44]. *Ex vivo* MRI scans were performed seven weeks after TBI. Normal rats were sacrificed and followed the same procedures and measurements as TBI rats.

#### 2) Ex-vivo imaging

We use a multi-shell imaging protocol using a Varian 7 Tesla MRI Scanner (Palo Alto, CA) with maximum applied gradient amplitude of 290 mT/m. HYbrid Diffusion Imaging (HYDI) [45] with 125 diffusion gradient directions in 5 shells is performed using a PGSE sequence with TR/TE/Δ/δ = 1500/40/18/10 ms and 5 averages. The number of directions in each shell is 6, 21, 24, 24, 50 with b-values of 360, 1440, 3240, 5760, 9000s/mm^2^, respectively, along with a reference T2 weighted B0 image. The FOV is 24 mm and slice thickness is1-mm, resulting in a 128×128 imaging matrix with 13 slices.

#### 3) Histological staining

The procedures to prepare brain sections were the same as previously reported [44]. Bielshowski and Luxol fast blue staining were used to identify reticular fibers (i.e. neurofibrils and neurofibrillary tangles) and myelin, respectively. For immunohistochemical staining, slides were placed in 20% silver nitrate in the dark, and ammonium hydroxide was added until the tissues turned brown with a gold background, and then sodium thiosulfate was added. The slides were stained for Luxol fast blue, washed in 95% alcohol and placed in lithium carbonate. Nuclei are colorless; myelin is blue and axons are black.

#### 4) Histological analysis

**Figure 6:**
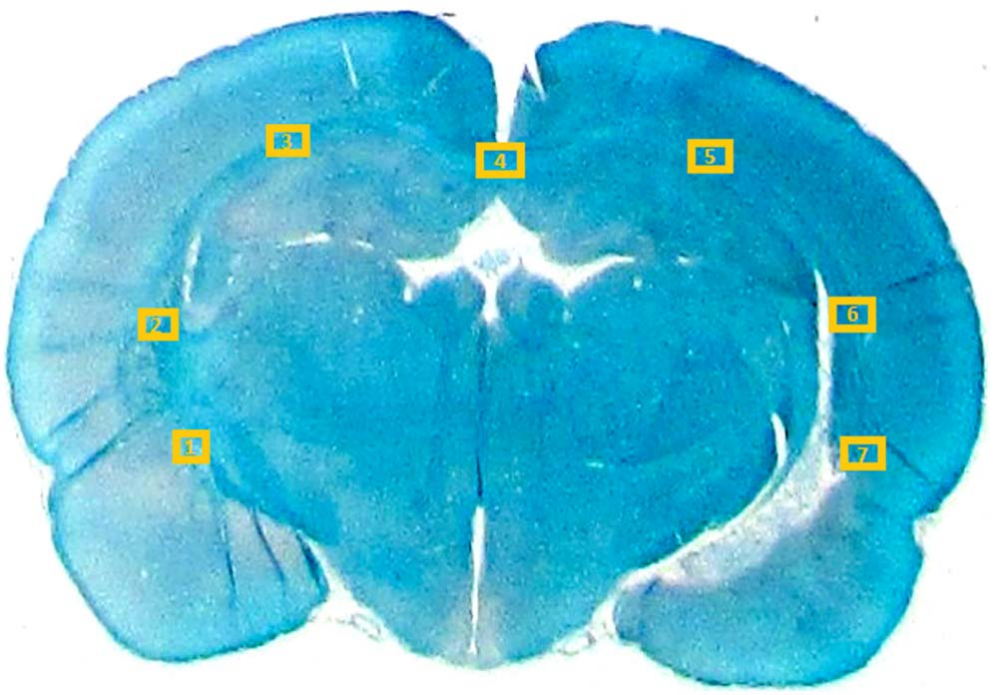
Illustration of the 7 ROIs considered in this study for taking microscopy images. All ROIs belong to the corpus callosum.

**Figure 7:**
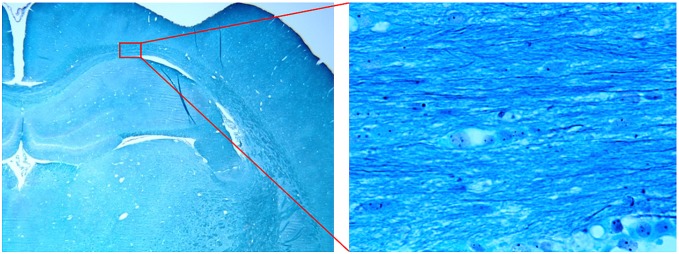
Typical microscopic images with 2x (left) and 100x (right) magnifications taken from ROI#5 of one of our cases. Nuclei are colorless; myelin is blue and axons are black in the stained sections.

Two sections of each brain were selected for taking light microscopy images with different magnification lenses (2x and 100x). The images were taken from seven parts of the corpus callosum (CC) as shown in Figure 6. If the tissue related to any of these ROIs is corrupted due to TBI or cutting process, the nearest region belonging to CC is considered for the photography. in Figure 7, typical images of ROI #5 for one of the TBI samples are shown.

The 100x magnified images were used for measurement of axon diameter and intra-axonal volume fraction. To measure the axon diameter, in each image at least 10 axons were selected visually and the number of pixels across the axons were counted. Since each pixel represents a 0.196 µm by 0.196 µm region in the actual tissue, axons thinner than this value may not be included in our calculations. To estimate the volume fraction, the images were converted to gray scale images. Then, after color inversion (to make the axons bright instead of dark) and histogram enhancement, the axons were extracted by thresholding the images using Otsu's method [46]. The soma and nuclei were also eliminated from the final binary image using morphological operations.

### f) Validation of the Proposed Model Using Histological and DWMRI Data

**Figure 8:**
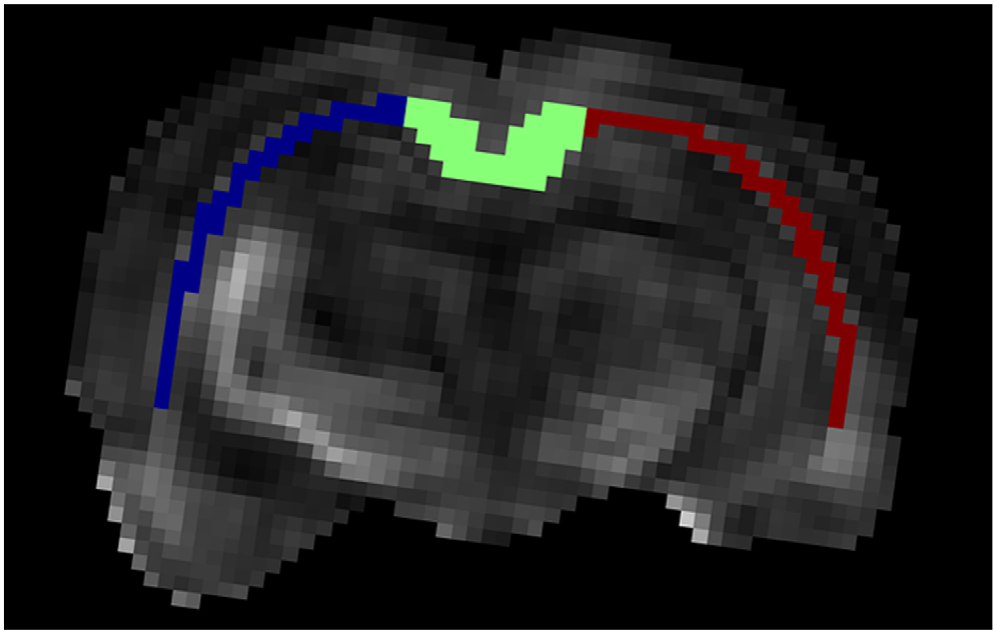
Illustration of the segmented regions of the corpus callosum fiber tract overlaid on the FA map. For each subject, the CC is segmented into three regions manually: left, right, and center. Our proposed model is applied to these regions and only the center part is used for validating the model using histological data.

As we described in Section (b), the brains were scanned before staining using the aforementioned protocol. The resulting images were smoothed using a 3×3 Gaussian filter to reduce the noise. For each brain, the corpus callosum (CC) fiber tract was segmented manually and divided into 3 parts: left, center, and right for comparison (see Figure 8). The remaining task for model validation is to find the corresponding regions between the MRI and histological images. This can be done by registration of each histological section to its corresponding T2-weighted image. However, even using a nonlinear registration algorithm, these two images will not be matched perfectly due to the significant intrinsic differences between these two modalities. For example, the MR imaging slice thickness is 1 *mm* while the histological section thickness is 6 *µm*. Moreover, there are some distortions and ruptures in the sections caused by the staining process. Therefore, a reliable way to find the corresponding regions is to do it visually. In our validation step, the center part of the corpus callosum of the normal cases is considered because this part is thick enough to assume that it is almost the same in the range of 1 mm. Also, correspondence of this region between MRI and histology can be established more reliably than the other regions. Needless to say, we can only validate the model with respect to the estimated structural parameters, i.e., the diameter and axonal volume fraction.

## Results and Discussion

### A) Histological Measures

**Figure 9:**
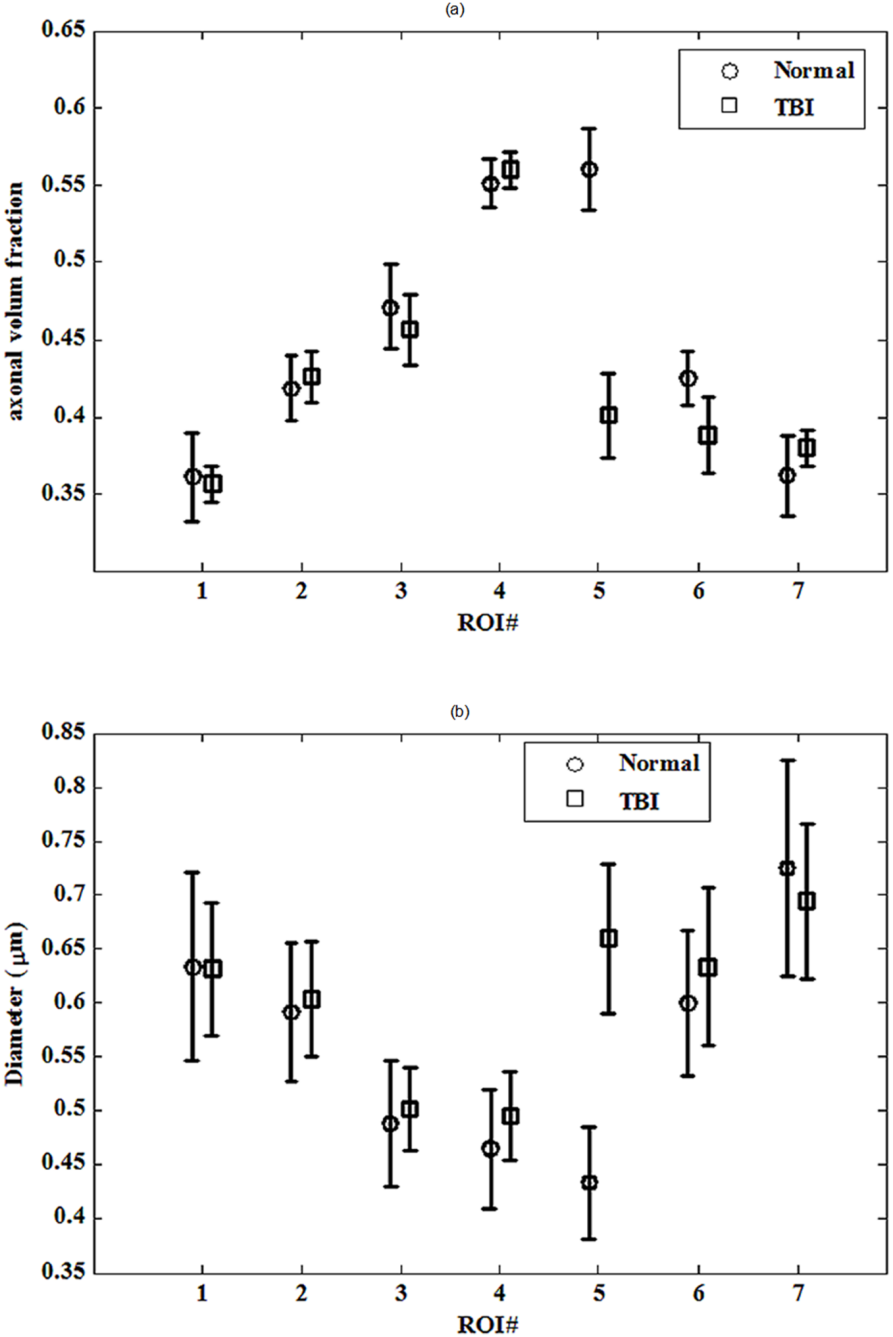
Error bar plots of the overall mean and standard error (SE) values of histologically measured parameters: (a) axonal volume fraction and (b) axon diameter for all cases. There is a distinct difference between normal and TBI rats in ROI#5 both for the diameter (with *p*-value of 5.7×10^−13^) and the axonal volume fraction (with *p*-value of 0.0033).

The measured values for diameter and axonal volume fraction of each ROI in the histological section for each case are shown in Tables 1 and 2, respectively. The corresponding plots of the diameter and axonal volume fraction versus ROI with error-bar are also shown in Figure 9. As seen, there is a significant difference between the normal and TBI rats in ROI#5 close to the TBI boundary, both for diameter (*p* = 5.7×10^−13^) and axonal volume fraction (*p* = 0.0033). In other words, the injury in ROI#5 after TBI has increased axonal diameters and reduced axonal volume fraction compared to the uninjured animals. As discussed in Section (d) of ‘Material and Methods’, these findings are consistent with the simulation results of our proposed model, where we found that by increasing WXR, the axonal volume fraction decreases while the axon diameter increases. Therefore, by considering the simulation results and histology findings, it can be claimed that the WXR increases in the injured brain.

**Table 1:** Measured values of the axon diameter for all cases.

	Case ID	ROI#1	ROI#2	ROI#3	ROI#4	ROI#5	ROI#6	ROI#7
**Normal** **cases**	TR01	0.56±0.10	0.56±0.12	0.59±0.13	0.53±0.11	0.45±0.12	0.58±0.14	0.58±0.07
	TR02	0.62±0.14	0.58±0.12	0.51±0.11	0.46±0.11	0.43±0.12	0.55±0.10	0.64±0.15
	TR03	0.77±0.24	0.57±0.11	0.42±0.10	0.47±0.10	0.40±0.08	0.53±0.07	0.64±0.12
	TR04	0.59±0.12	0.64±0.15	0.47±0.09	0.40±0.09	0.45±0.09	0.69±0.14	0.88±0.20
**TBI cases**	TR76	0.68±0.22	0.57±0.10	0.48±0.11	0.49±0.14	0.99±0.29	0.97±0.38	0.67±0.17
	TR77	0.62±0.16	0.52±0.09	0.45±0.11	0.51±0.15	0.69±0.11	0.63±0.22	0.65±0.15
	TR79	0.66±0.22	0.66±0.13	0.55±0.11	0.50±0.11	0.57±0.13	0.60±0.15	0.96±0.38
	TR82	0.57±0.18	0.45±0.11	0.55±0.15	0.49±0.11	0.55±0.13	0.52±0.15	0.62±0.17
	TR83	0.60±0.13	0.58±0.12	0.53±0.10	0.56±0.15	0.53±0.18	0.49±0.07	0.64±0.15
	TR86	0.81±0.30	0.60±0.16	0.47±0.07	0.47±0.10	0.68±0.21	0.60±0.10	0.69±0.21
	TR88	0.60±0.16	0.67±0.22	0.44±0.12	0.43±0.12	0.58±0.16	0.60±0.14	0.64±0.12
	TR89	0.60±0.21	0.55±0.14	0.52±0.08	0.51±0.12	0.78±0.20	0.66±0.15	0.74±0.23
	TR90	0.58±0.16	0.74±0.19	0.51±0.10	0.58±0.14	0.61±0.16	0.61±0.14	0.68±0.23
	TR91	0.63±0.13	0.56±0.19	0.58±0.15	0.43±0.10	0.48±0.18	0.57±0.12	0.67±0.09

**Table 2:** Estimated values of the axonal volume fraction for all cases.

	Case ID	ROI#1	ROI#2	ROI#3	ROI#4	ROI#5	ROI#6	ROI#7
**Normal** **cases**	TR01	0.40	0.44	0.44	0.54	0.61	0.42	0.46
	TR02	0.28	0.42	0.42	0.52	0.60	0.44	0.36
	TR03	0.36	0.44	0.56	0.60	0.58	0.43	0.37
	TR04	0.40	0.39	0.47	0.55	0.48	0.41	0.31
**TBI cases**	TR76	0.32	0.45	0.43	0.57	0.35	0.31	0.36
	TR77	0.38	0.42	0.59	0.63	0.23	0.27	0.34
	TR79	0.32	0.38	0.33	0.43	0.31	0.34	0.35
	TR82	0.27	0.40	0.36	0.53	0.45	0.44	0.40
	TR83	0.43	0.47	0.54	0.56	0.56	0.51	0.39
	TR86	0.36	0.54	0.55	0.59	0.41	0.54	0.35
	TR88	0.33	0.34	0.45	0.53	0.27	0.25	0.26
	TR89	0.33	0.45	0.45	0.52	0.29	0.30	0.34
	TR90	0.36	0.31	0.47	0.58	0.49	0.38	0.39
	TR91	0.37	0.45	0.48	0.56	0.57	0.49	0.46

### B) Validation of the Proposed Model Using MRI and Histology Data

Table 3 shows the estimated values of the diameter, 2*R*, and axonal volume fraction, *v*, for the center part of the CC of normal cases using our proposed model. By comparing this table to the histological values shown in Tables 1 and 2, especially ROI#4, it can be seen that *v* is underestimated (about 30%) while R is overestimated (about 20%). However, the results are consistent because the consistency analysis between the results of histology and the proposed model reveals that the intra-class correlation coefficients within 95% of confidence interval are 0.970 (*p* = 0.008) and 0.933 (*p* = 0.026) for the diameter and volume fraction of the axons, respectively.

**Table 3:** Estimated values of the diameter, 2*R*, and axonal volume fraction, *v*, for the center part of the corpus callosum in normal cases, estimated by fitting our proposed model to the DWI data.

	2*R* (µm)	V
TR01	0.60±0.13	0.38±0.07
TR02	0.54±0.11	0.36±0.08
TR03	0.52±0.10	0.41±0.09
TR04	0.49±0.09	0.38±0.06

The results are consistent with the measured values shown in Tables 1 and 2. The intra-class correlation coefficients within 95% of the confidence interval are 0.970 (*p* = 0.008) and 0.933 (*p* = 0.026) for the diameter and volume fraction of the axons, respectively.

The observed differences between the results of the model and the histological measurements can be caused by several factors. These factors generally originated from the limitations in modeling (simplifying assumptions like parallel, same size axons), imaging (like noise), and also histology staining process. The latter factor includes the inevitable unwanted incidences which may occur during cutting and staining such as corruption, contraction, or expansion of the tissue. On the other hand, measurement of the radius and axonal volume fraction has its own limitations. For instance, the pixel size on the 100x-magnified images is 0.196 *µ*m which imposes an uncertainty of about 0.2 *µ*m to the values of measured axon diameter. Moreover, the thickness of the sliced sections (which is 6 *µ*m) has not been accounted for measuring the volume fraction.

### C) Application of the Proposed Model to Discriminate TBI Tissues

**Figure 10:**
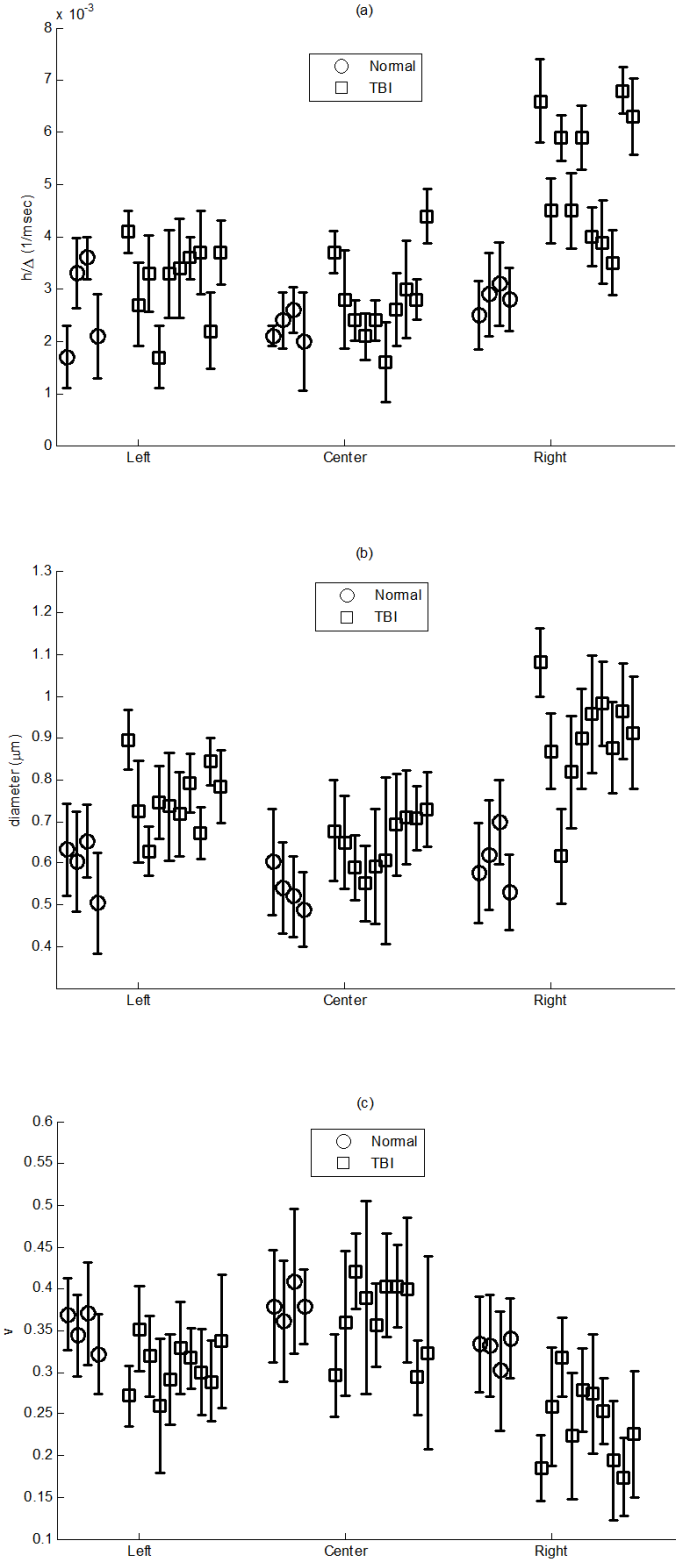
The error bar plots of mean and STD values of the estimated parameters: (a) *h*, (b) 2*R*, and (c) *v* for all normal (blue) and TBI (red) cases using the proposed model. As seen, there is a clear contrast, with *p*-value of 0.001, between the injured (right) and normal (left) parts of the CC, especially for the map of the parameter *h*, which is related to the water exchange rate (WXR).

To show the capability of the model to discriminate between the injured and normal tissues, three parts of the CC were segmented manually as depicted in Figure 8 and investigated using the proposed model. In Figure 10, the error-bar diagrams (mean with STD values) of the estimated parameters, *h/Δ*, 2*R*, and *v* in different ROIs are plotted for all cases including normal and TBI rats. There is a clear contrast with *p*-value of 0.001 between the injured (right) and normal (left) parts of the CC, especially for the map of *h/Δ* parameter (related to the water exchange rate, WXR) which significantly increases in the TBI boundary compared with that in the controls. Also, as expected, the diameter increases and the axonal volume fraction decreases in the injured fibers, which is consistent with our simulation results.

The proposed model can also be employed to investigate other diseases affecting the white matter fiber tracts such as multiple sclerosis (MS). Especially, the estimated values of *h* in the fiber tracts close to or within the damaged area may be used as a quantitative measure to show the progress of the disease. For example, this parameter may demonstrate the level of demyelination since there is a meaningful relation between the myelin sheath and the exchange rate. Therefore, as the proposed model can be applied to the data acquired using clinical scanners, our model provides a new insight that may be relevant to clinical diagnosis and treatment of neurological diseases.

Although the methods presented in this work are based on high b-value DWMRI, it is worthy to discuss the effect of changing micro-structural parameters on the DTI parameters such as fractional anisotropy (FA) and mean diffusivity (MD). Generally, increasing WXR may decrease FA while increase MD. On the other hand, if the intra-axonal space increases, the diffusion inside the axons becomes less ‘restricted’, however, the diffusion outside of the axons becomes more ‘hindered’. Therefore, if the hindered part is the dominant part of the diffusion signal (which is true in common DTI protocols), by increasing the intra-axonal space the FA is increased while the MD is decreased, and vice versa. It should be implied that the true effect of variation of the axonal space on the FA and MD values is dependent on several factors such as imaging parameters and tissue specifications.

Brain injury, such as TBI, remains a leading cause of mortality and disability among children and young adults. Current research in brain injury has been restricted to acute neuroprotection treatment with a short treatment window [47]–[49]. In these investigations, functional recovery after TBI may be driven by neuronal remodeling. Currently, investigation of neuronal remodeling after brain injury has been dominated by traditional DTI such as FA and fiber tracking [50]–[55]. However, conventional DTI produces an anomalous result, showing an overall lowering of FA despite the presence of highly-oriented tissue [3], [44], [56]–[60]. Currently, there are few published studies to target the issue related to low FA in areas with highly-oriented tissue [44], [57], [58], [60], [61] and no investigation has been published in white matter remodeling after brain injury, such as TBI, using MRI axonal permeability and diameter. Our data demonstrated that physiological measurable parameters, MRI axonal permeability, diameter, and density are sensitive in detecting axonal changes after TBI and could be very useful in the evaluation of axonal damage and remodeling after neurological diseases, such as TBI, stroke, and MS.

## Conclusion

In this work, a new analytical diffusion model was proposed for estimating micro-structural (axon diameter and volume fraction) and diffusivity (diffusion coefficients as well as water exchange rate) parameters of white matter fiber tracts using DWMRI. Therefore, one of the specifications of the proposed model is that it includes the absorbing coefficient of the intra- axonal space. This coefficient is linearly related to the water exchange rate (WXR), which has not been fully investigated in previous calculations. Moreover, the proposed model does not require short gradient pulse (SGP) approximation and thus can be applied to the data acquired using common PGSE sequences in a clinical scanner. The simulation results using Markov Random Walk showed that the included parameter, *h*, in the model has a significant, proportional relation to the actual water exchange rate, WXR. Moreover, the simulations revealed that by increasing WXR the axon volume fraction decreases while the diameter increases. Consistent with our model, histological analysis of TBI brain rats revealed that in the injured tissues, the volume fraction reduced and the axon diameter increased. Therefore, according to these findings, it can be concluded that the WXR increases in injured tissues, which is reasonable, because the cell membrane, especially the myelin sheath, is damaged. The estimated values by applying our proposed model to the MRI data of normal rats were relatively comparable to their corresponding histological measurements. Nevertheless, the observed differences can be caused by several factors originating from the limitations in modeling, imaging, and also the histology experiment. As shown in this work, the proposed model can be used to discriminate TBI and normal tissues with respect to the estimated parameters, especially WXR. One of the limitations of the proposed model, as usually exists in other similar models, is that it can only be applied to specific regions of the white matter where the axons are parallel and the same size. This issue can be addressed in the future by adding more compartments to the model as well as improving the MRI data acquisition.
